# Facial attractiveness does not modify the perceived trustworthiness of ethnic minority men

**DOI:** 10.1038/s41598-024-78291-9

**Published:** 2024-11-07

**Authors:** Joshua Hellyer

**Affiliations:** Mannheim Centre for European Social Research, University of Mannheim, 68131 Mannheim, Germany

**Keywords:** Trustworthiness, Discrimination, Migration history, Physical attractiveness, Germany, Human behaviour, Psychology and behaviour

## Abstract

Immigrants, and particularly immigrant men, are often stereotyped as untrustworthy in European societies. However, little research has examined how stereotypes of characteristics other than ethnicity might impact natives’ perceptions of the trustworthiness of immigrants. Here, I test whether facial attractiveness, a trait associated with a variety of positive stereotypes, might modify ethnic biases in trustworthiness perceptions. I vary facial attractiveness and ethnicity using photo and name stimuli presented in a hypothetical “lost wallet” vignette, in which respondents assess the likelihood of the pictured man returning their lost wallet. Results from an German online panel survey indicate that while attractiveness has a modest positive effect on perceived trustworthiness, the value of attractiveness does not differ between ethnic majority German men and men with a Turkish migration background. Rather, the largest differences in the perceived trustworthiness of Turkish-origin men are found between respondents with inclusionary and exclusionary immigration attitudes, with inclusionary respondents reporting that Turkish-origin vignette persons are more trustworthy than ethnic majority German vignette persons. These results suggest that physical attractiveness does not act as a substantial moderator of ethnic biases in trustworthiness perceptions, but that immigration attitudes are highly relevant.

## Introduction

In contemporary societies, we are often faced with situations where we must place trust in unknown others. In many of these interactions, we must quickly assess a person’s trustworthiness based on only a first impression, which is often heavily influenced by the appearance of a person’s face^1–3^. Whether or not these judgments are accurate^4^, people seem to believe that they can infer the trustworthiness of an interaction partner from their appearance^5^ and may rely more on facial cues than information about past performance^6^. Beyond behavior in behavioral tasks, these instantaneous judgments can have serious consequences in a variety of real-world social interactions including partner selection, voting behavior, and criminal justice decisions^7^.

A person’s face can be used to glean a variety of information, potentially including such traits as ethnicity and gender identity, which might factor into a potential trustor’s decision about whether or not to trust. In the language of status characteristics theory, traits like these are considered ascribed status characteristics with levels that are differentially valued^8^. These particular traits are also “diffuse,” meaning that they are associated with general expectations about performance in a variety of tasks, as opposed to a characteristic like language ability that may only affect performance in specific tasks. Thus, stereotypes about the value or prestige of a certain diffuse status characteristic may influence a person’s perceived trustworthiness. For example, if members of minority ethnic groups are seen as lower in status than the local ethnic majority, they may also be seen as less likely to behave prosocially in a trust situation. However, empirical evidence for this phenomenon is mixed and may depend on the specific groups and context being studied^9,10^.

From this perspective, one might expect that immigrants, a broadly defined out-group that commonly faces derogation in many societies, would be seen as untrustworthy. Psychological research on perceptions of outgroup faces (not necessarily immigrants, per se) would support this assertion^11,12^. However, experimental evidence on the perceived trustworthiness of immigrants is mixed^13–16^. One possible explanation for these findings could be that considerable heterogeneity exists within the broad category of “immigrants,” such that certain sub-groups of immigrants are considered particularly untrustworthy while others are not^17^. For example, recent evidence from Germany finds that men with a migration history are seen as particularly untrustworthy, while women with a migration history are not^18^.

Another possible explanation is that considerable heterogeneity exists in natives’ attitudes toward immigrants, which might differentially influence their perceptions of immigrants’ trustworthiness. While research on immigration attitudes and trustworthiness perceptions is limited, previous research on race in the U.S. has found that implicit race attitudes shape trustworthiness perceptions, with more biased individuals finding Black faces less trustworthy^19,20^. Likewise, we might expect that people with more exclusionary attitudes toward immigrants might find immigrants more threatening and less trustworthy. While recent work finds that preference for natives does not have a significant effect on trustworthiness perceptions of immigrants^15^, this question has not yet been tested in Germany, or with broader measures of immigration attitudes.

This article seeks to understand how these (gendered) ethnic stereotypes about trustworthiness might also be affected by another highly salient trait: physical attractiveness. Following Dion, Berscheid and Walster’s seminal hypothesis of that “what is beautiful is good”^21^, attractiveness may be a particularly important predictor of perceived moral character^22^. Due to the fast and automatic nature of attractiveness judgments^23^, it has been suggested that people use attractiveness as a heuristic to assess the inherently invisible trait of trustworthiness^24^. This would help to explain results from behavioral games finding that more attractive people are generally thought to be more trustworthy^25–28^.

Why might attractiveness affect ethnic stereotypes about trustworthiness? The positive stereotypes associated with physical attractiveness differ substantially from the often negative stereotypes associated with ethnic minority groups. As suggested in the Stereotype Content Model, stereotypes are thought to contain information about two dimensions: warmth and competence^29^. The warmth dimension includes assessments of sociability but also of morality, including perceptions of trustworthiness. Germans rate Turks as lower in warmth than co-ethnics^30^, and gendered stereotypes of criminality, aggression, and misogyny among men from Muslim-majority countries also indicate negative stereotypes about warmth^31^. Attractiveness may provide a signal of warmth or sociability that counteracts some of these negative stereotypes^32^, making attractive Turkish-origin men seem more trustworthy than their less attractive counterparts. This would align with previous research that suggests that attractiveness may be a signal of atypicality for members of minority groups^33,34^, which may be of particular benefit to those in groups facing negative stereotypes, such as Muslim immigrant men. This suggests that attractiveness may benefit men of Turkish descent more than ethnic majority German men.

Based on these findings, I develop several hypotheses about the effects of facial attractiveness and ethnicity on perceptions of trustworthiness. First, I expect to find that highly attractive faces are seen as more trustworthy (H1). In terms of ethnicity, I expect that members of one’s ethnic in-group will be seen as more trustworthy. Varying two signals of ethnicity, I expect that either signal of belonging to an ethnic minority group (phenotype in H2 or name in H3) will be associated with lower perceived trustworthiness. Finally, I explore the intersection of facial attractiveness and ethnicity, expecting that attractiveness will have a larger positive effect for ethnic minority men (H4).

To assess whether the size of the “beauty premium” in trustworthiness perceptions varies across ethnic groups, I use a vignette experiment embedded within the German Internet Panel (GIP), a longitudinal study with a large sample that is representative of the German population^35^. As a measure of the perceived trustworthiness of a vignette person, I use the “lost wallet question,” a tool previously used to measure relational trust^36,37^. The term “relational trust” relates to trustworthiness expectations in a two-party framework where one party has an incentive to profit from (or here, withhold assistance from) the other^38^. In the lost wallet question, the respondent is asked to imagine that they have lost their wallet and that it was found by the person described in the vignette. They are then asked to assess how likely they think it is that their wallet will be returned based on the information they are shown about the person. Thus, the measure captures trustworthiness perceptions of a specific person in a specific scenario, improving upon other measures of trust which ask about expectations of a vaguely defined group of “most people”^39^. Comparing responses between 16 male vignettes that vary in terms of signals of ethnicity (names and phenotypes) and facial attractiveness allows for a comparison of the size of the beauty trust premium across ethnic groups.

## Methods

This article uses data from wave 70 of the German Internet Panel (GIP), a large longitudinal survey of people between the ages of 16 and 75 who live in Germany. Respondents were recruited offline. Initially, respondents from randomly selected neighborhoods were selected via random route sampling and invited to participate in person. In later waves of recruitment, respondents were randomly selected from registry data and invited by mail^35^. Respondents answer surveys on a variety of topics every two months; wave 70 was fielded in March 2024.

Of the 3,681 respondents who participated in this wave, 1,937 were randomly selected into participating in this experiment. 34 respondents were excluded from this analysis due to missing values for gender, age, and/or the outcome variable. Because respondents’ assessment of the vignette person may also be influenced by their own ethnicity, or more specifically whether the vignette person is a member of their ethnic in-group^11,12^, I attempt to restrict the sample to ethnic majority native Germans. While no information about ethnicity or migration background is provided in recent waves of the GIP, I use information about respondents’ citizenship as the best available proxy. I thus exclude 109 respondents who do not have exclusively German citizenship, leaving me with a final analytical sample of 1,794. As shown in the descriptive statistics provided in the Supplementary Information (Table S1), the selected sample and the “unselected” sample (those assigned to another experiment) do not differ significantly on most demographic variables used in this analysis. However, once exclusions are applied, respondents selected for this experiment are slightly more likely to have passed a matriculation examination (*Abitur*), 41% to 38%.

The design of this study was pre-registered on OSF [https://osf.io/2djpa]. Each respondent answered one lost wallet vignette containing a headshot-style photo of the person described as well as the following text (translated from German): “[NAME] is 25 years old and grew up near your current place of residence. Imagine that you lose your wallet (containing ID and documents) on the street and [NAME] finds it. What do you think: in this situation, will you get your wallet back?” Responses are given using a 5-point Likert scale ranging from “I will definitely not get it back” to “I will definitely get it back.” Given that all vignette persons are said to be raised in the respondents’ area, the profiles with migration background should be interpreted as at least 1.5 generation, if not second-generation, immigrants, and not as recent arrivals or tourists. Respondents were randomly assigned to one of sixteen vignettes that vary only in the name and photo shown. However, these two stimuli alone signal several factors, specifically gender, ethnicity, and facial attractiveness.

### Stimuli

**Fig. 1:**
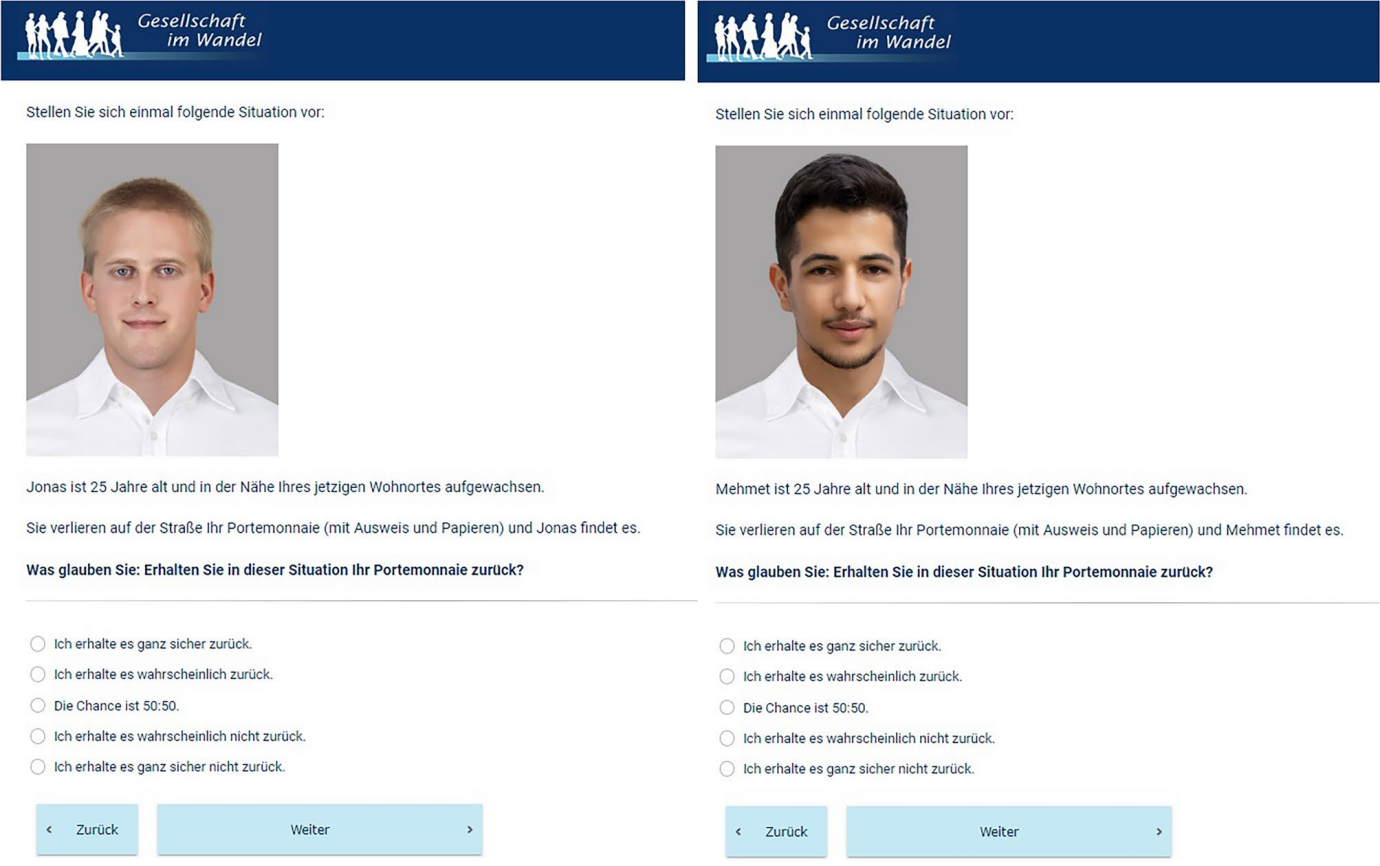
Example vignettes in German low-attractiveness condition (left) and Turkish high-attractiveness condition (right)^63^. Face imagery sources: left, Chicago Face Database^42^ and right, DeZIM Picture Database: Faces^41^.

Face images used as stimuli in this project have been collected from several sources. For Turkish faces, I use a subset of photos from the Bogazici Face Database, a collection of photos of Turkish undergraduate students^40^, as well as some photos of Germans with Middle Eastern or North African (MENA) migration history from the DeZIM Picture Database: Faces^41^. For German faces, I use photos of white German men from the DeZIM database and photos of white American men from the Chicago Face Database^42^. All of the people pictured have explicitly consented to having their photos used in scientific research. All selected faces show a similar expression: neutral or slightly smiling but with no teeth visible. Given that happy faces are seen as more trustworthy^43^, this selection minimizes the potential for facial expression to significantly affect results. The selected photos have been edited such that all photos have matching clothing and appear against the same background, as shown in Fig. 1. The edited photos have been rated on their attractiveness, ethnic typicality, perceived social class, and trustworthiness (among other features) by a large sample of German adults from an online access panel (N = 1,125).

Some photos that were rated about equally likely to be Turkish or German will be used in both name conditions, separating the effects of two different cues of ethnicity: the photo and the name. This allows me to test whether visual and name cues have different effects, and whether having an ethnically ambiguous appearance further impacts perceptions of trustworthiness.

I used these ratings, as well as a set of names rated on the same characteristics by another sample of German adults (N = 800), to choose combinations of name and photo that clearly convey gender (male), ethnicity (German or Turkish), and level of facial attractiveness (low or high). To satisfy these conditions, I selected the names Jonas for ethnic majority German men and Mehmet for men of Turkish descent. I also chose photos in order to maximize the difference in attractiveness between groups while minimizing differences within groups, yielding a highly attractive group (mean rating 6.27 out of 11) and a low-attractiveness group (mean rating 4.90).

Altogether, the survey uses 16 different vignettes in a full factorial design. This includes two different photos for each of the 8 unique combinations of attractiveness (high or low), ethnicity (white German or Turkish), and ethnic ambiguity (ambiguous or unambiguous phenotypes). Using two photos helps to ensure that effects are based on the vignette person’s characteristics and not driven by possibly idiosyncratic perceptions of a single photo.

### Ethical approval

This study design was approved by the University of Mannheim Ethics Commission (EK 04/2023). Informed consent was obtained from all participants of the German Internet Panel, and all participants were reimbursed for their time. The study design complies with German and European law as well as the ethical guidelines of the German Sociological Association.

### Statistical analysis

As pre-registered, I first separately estimate the effect of high facial attractiveness on trustworthiness perceptions using OLS models without any additional control variables. Similarly, I estimate the effect of (Turkish) ethnicity on trustworthiness perceptions, separately estimating the effect of a Turkish name among only those vignettes in the ambiguous condition (i.e., those with photos also used in the German name condition) and the effect of a Turkish name and phenotype among vignettes in the unambiguous condition. I then estimate the interaction between attractiveness and (Turkish) ethnicity. Next, I estimate all five of these models with additional control variables that may impact respondents’ perceptions of trustworthiness, including respondents’ gender (male or female), age (born before or after 1970), educational attainment (*Abitur* or no *Abitur*), employment status (in work or training or not), marital status (living with partner or not), and region (East or West) [In a deviation from the pre-registration, community size (rural or urban) is not included as a control variable due to missing geographic information for many respondents. Analyses including geographic variables for the sample with valid postal codes are reported in the Supplementary Information, Table S7]. Statistical analyses were conducted using Stata 17^44^.

## Results

**Fig. 2:**
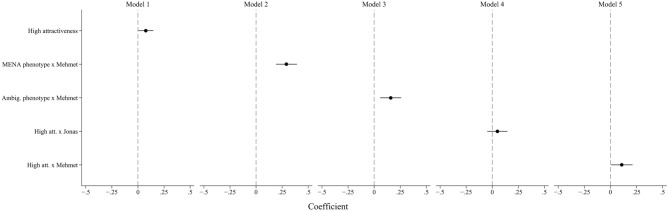
Coefficients and 95% confidence intervals from multivariate regression analysis (OLS) without control variables, full sample.

Results from regressions without control variables are shown in Fig. 2, and in tabular form in the Supplementary Information (Table S2). As hypothesized (H1), I find a positive effect of high attractiveness on trustworthiness perceptions, although this effect is relatively small ($p =$ 0.037, Model 1). More surprising are the results for signals of Turkish ethnic background (Models 2 and 3), both of which are associated with significant increases ($p =$ 0.000 and $p =$ 0.002, respectively) in perceived trustworthiness compared to ethnic German vignettes. This effect appears to be particularly strong among vignettes with unambiguous MENA phenotypical and name signals (Model 2) compared to those with an ambiguous MENA-European phenotype and the same name (Model 3). These findings contradict my expectations in Hypotheses 2 and 3, in which I expected to find a penalty for both signals of ethnic minority group membership.

Finally, in Models 4 and 5, I examine the interplay of facial attractiveness and ethnicity. Despite the overall positive effect of high attractiveness, this effect is only statistically significant for Turkish-origin vignettes ($p =$ 0.325 for Jonas and $p =$ 0.039 for Mehmet). While this finding points in the expected direction, i.e. that attractiveness may benefit ethnic minority men more than ethnic majority men, analyses shown in Supplementary Information, Table S4 show that the value of facial attractiveness does not vary between ethnic groups ($p =$ 0.399 without controls). Thus, I find no support for Hypothesis 4.

As shown in the Supplementary Information, Fig. S1 and Table S3, adding control variables does not substantially affect the direction or significance of these results. This demonstrates that these results largely cannot be explained by differences in gender, educational attainment, employment status, marital status, or region. Rather, these variables have effects in the expected directions across treatment categories, such that highly educated, female, older, West German, and partnered people generally assign higher trustworthiness ratings. In a separate exploratory analysis presented in the Supplementary Information, Table S7, I also add two control variables related to respondents’ location of residence (for respondents who reported a valid postal code, $N =$ 1,370): the share of migrants in their local area and whether they live in an urban or rural community (defined as a community with fewer than 10,000 residents). These variables, taken from 2011 census data^45^ (the most recent available with respect to migrant populations), do not exert a significant impact in most models (although respondents in urban areas may be slightly less trusting), and the overall patterns presented above remain unchanged.

Because the outcome variable studied here is ordinal, I also perform an exploratory analysis in which I estimate all models including controls as ordered logistic models. As shown in the Supplementary Information, Table S5, the same patterns emerge in the results of these ordinal models, suggesting that the choice of estimator is not substantially driving the results. I also perform additional exploratory analyses in the Supplementary Information, Table S6, that use the mean pre-rated attractiveness value rather than a binary classification of facial attractiveness and focus only on vignettes with unambiguous phenotypes. In these models, the effect of facial attractiveness loses significance ($p =$ 0.732 in Model 1). Model 2 suggests that facial attractiveness may have a larger effect for Mehmet, but as in the original analyses, the difference in the effect size of facial attractiveness does not significantly differ between Jonas and Mehmet ($p =$ 0.569 in Model 3), even when adding controls ($p =$ 0.498 in Model 4). Despite the lack of significant results, the effects are largely in the same direction as previous models, and do not change the conclusion that the effects of facial attractiveness do not differ by ethnicity.

### Exploratory analysis: heterogeneous effects

While results regarding attractiveness largely conform to theoretical expectations, the finding of a substantial trust premium for ethnic minority men is wholly unexpected. To explore this in greater detail, I conduct several exploratory analyses to determine whether these overall results might mask heterogeneous results based on other uncontrolled respondent characteristics.

Even though all vignette persons are described as longtime residents of Germany (i.e., they grew up in the area where respondents live), immigration attitudes may be a particularly important characteristic driving attitudes toward vignette persons with a migration background. Respondents with exclusionary immigration attitudes may have stronger and more negative stereotypes of people with migration background than more inclusionary respondents do. Therefore, I subdivide the sample into two groups based on respondents’ immigration attitudes. In the same wave of the GIP (after, but not immediately following the trust vignette), respondents answered three questions about immigrants in Germany which have been previously used to measure immigration attitudes in the European Social Survey^46,47^. They were asked whether immigration is overall good or bad for the economy, whether immigrants generally undermine or enrich cultural life, and whether it is good or bad that immigrants have the right to come to Germany. Responses were collected using a scale from 0 to 10. I recoded these questions so that positive attitudes were consistently associated with larger values, then calculated the mean of the three responses to create an index of immigration attitudes. I divide the sample into two groups, an “inclusionary” group of those with a score higher than 4 (n = 1,294, 79% of the total sample) and an “exclusionary” group of those with a score of 4 or lower (n = 344, or 21%).

**Fig. 3:**
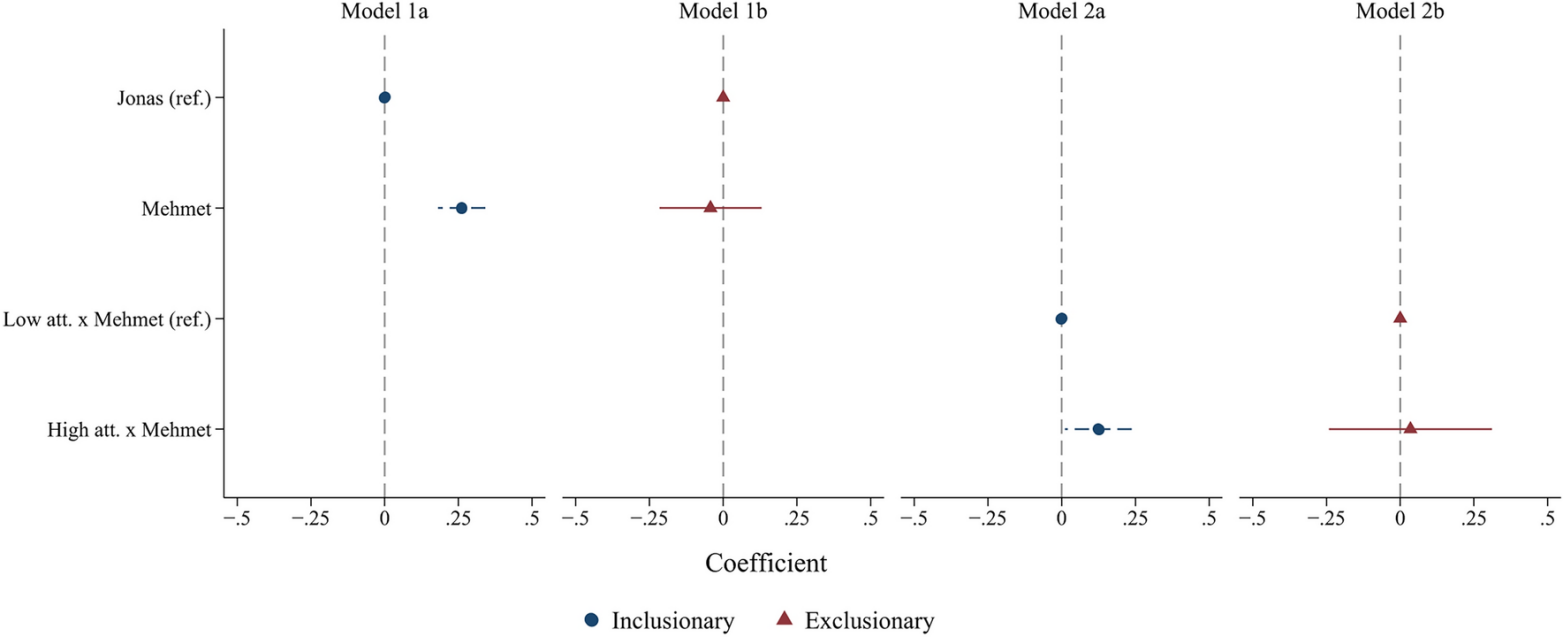
Coefficients and 95% confidence intervals from multivariate regression analysis (OLS) without control variables. Sample divided into “inclusionary” (index score > 4, n $=$ 1,376) and “exclusionary” respondents (index score $<=$ 4, n $=$ 357).

In Fig. 3 (and Table S8 in the Supplementary Information), I show results for the ethnicity-related items for each of these two subgroups. Because results are broadly similar between MENA and ambiguous phenotypes, I report results only by name (Mehmet vs. Jonas). As shown in Models 1a and 1b, the positive effect of a Turkish name on trustworthiness perceptions is only found among the inclusionary respondents ($p =$ 0.000), while the most exclusionary respondents do not significantly differentiate between German and Turkish vignettes ($p =$ 0.626). As shown in the Supplementary Information, Table S10 (Models 1 and 2), the difference in the effect of a Turkish name between inclusionary and exclusionary respondents is statistically significant ($p =$ 0.002 without controls). Additionally, coefficients reported in Table S8 indicate that exclusionary respondents rate all vignettes as less trustworthy than do inclusionary respondents, suggesting differences in overall trust behavior between these groups. Turning to the interaction of attractiveness and ethnicity, Models 2a and 2b show that inclusionary respondents find more attractive Turkish vignette persons to be more trustworthy relative to their less attractive counterparts ($p =$ 0.031), while exclusionary respondents do not differentiate based on attractiveness ($p =$ 0.806). This suggests that facial attractiveness cannot close the ethnic “trust gap” for these exclusionary respondents, but it may serve as an additional signal of trustworthiness for inclusionary respondents rating Turkish profiles.

While the vignettes here do not include any signal of religion or religiosity, respondents may assume that a person with Turkish migration background is Muslim. As a measure of respondents’ attitudes toward Islam, I examine the results of an experiment fielded in the same wave of the GIP (after but not immediately following the trust vignette) that measures respondents’ support for building a mosque in their state’s capital city. While the experiment varies textual and visual descriptions of the planned mosque, I pool the results and group the four response categories into a single binary indicator of support (n $=$ 734) or opposition (n $=$ 806) to the construction of a mosque regardless of experimental condition. Results dividing the sample into these two groups are shown in the Supplementary Information, Table S9. These results echo those in Fig. 3 but with less extreme division between the groups, with pro-mosque respondents reporting higher perceived trustworthiness for Turkish vignettes ($p =$ 0.000) and anti-mosque respondents reporting no significant difference between Turkish and German vignettes ($p =$ 0.227). As shown in the Supplementary Information, Table S10 (Models 3 and 4), the difference between pro-mosque and anti-mosque respondents’ ratings of Turkish-origin vignettes is statistically significant ($p =$ 0.000 without controls). Here, attractiveness has no significant effect on trustworthiness perceptions for Turkish vignettes ($p =$ 0.136 for pro-mosque respondents and $p =$ 0.604 for anti-mosque respondents), but effects run in the same direction as in Fig. 3.

I also examine the effect of respondent gender. Considering the effect of gender is important in analyses of physical attractiveness, as (heterosexual) men and women may respond differently to the attractiveness of male vignette persons. Furthermore, women are more motivated to control prejudice, which may lead them to report more socially acceptable responses^48^. However, there are no substantial gender differences between men and women in these results (Supplementary Information, Fig. S2 and Tables S11 and S12), and while the trends resemble the overall results in Fig. 2, most coefficients are not statistically distinguishable from zero, with the exception of a large and significant positive effect of a MENA-phenotype Mehmet for both male and female respondents ($p =$ 0.001 and $p =$ 0.000, respectively), and a significant positive effect ($p =$ 0.002) of ambiguous Mehmet for female respondents only.

Finally, I consider the effect of socioeconomic status, which may influence ethnic differences in perceptions of trustworthiness. Previous research has found that the social status of both trustor and trustee may affect trustworthiness perceptions^10^. This may be particularly problematic when examining differences related to ethnicity, given that people with Turkish migration background have on average lower educational attainment and income than ethnic majority Germans^49^. As natives are more likely to discriminate against ethnic minorities who are lower in class^50,51^, the results presented so far may conflate ethnic and class-based discrimination. To disentangle these effects, I first examine whether the effects of ethnicity on trustworthiness perceptions vary with respondents’ educational attainment, specifically whether or not they received the *Abitur*, as a proxy for overall socioeconomic status. As shown in the Supplementary Information, Table S13, vignettes with the name Mehmet are seen as more trustworthy by respondents regardless of their educational attainment, even when adding control variables. To estimate the interaction between respondents’ socioeconomic status and the perceived status of the vignette person, I also estimate models that include ratings of each vignette person’s social class from the pre-test (N $=$ 1,125). In Table S14 in the Supplementary Information, I show that pre-rated social class has no significant effect on trustworthiness perceptions (in models without controls, $p =$ 0.642 for respondents without *Abitur* and $p =$ 0.261 for those with *Abitur*), and the name Mehmet is still associated with higher perceived trustworthiness. From these analyses, I conclude that perceptions of class do not seem to underlie respondents’ perceptions of Turkish-origin vignette persons.

## Discussion

Results from a vignette experiment confirm previous results about the link between attractiveness and trustworthiness: more attractive profiles are rated as more trustworthy, although the effect size is relatively small. However, despite theoretical expectations, the size of the beauty premium in trustworthiness perceptions does not seem to vary between native ethnic majority German men and men of Turkish descent. While prior research suggested that attractiveness might signal atypicality, reducing negative stereotypes of stigmatized groups like Turkish immigrant men, that does not seem to be the case with respect to trustworthiness perceptions in the German context. Finally, I find that while respondents with exclusionary immigration attitudes rate members of both ethnic groups as roughly equally trustworthy, those with inclusionary attitudes rate Turkish vignettes as more trustworthy than German vignettes.

This last finding is especially striking, given that previous research has largely found that immigrants, and particularly immigrant men, are perceived to be either less trustworthy^16,18^ or about equally as trustworthy as natives^13–15,52^. Additionally, immigrant men of Middle Eastern descent are often stereotyped as particularly untrustworthy in Europe^31^.

One of two main possibilities seems likely to explain this phenomenon. The first possibility is that the respondents truly found the Mehmet vignettes more trustworthy than the Jonas vignettes. Perhaps the specific vignette information presented here influenced perceptions of the vignette people in ways that were not anticipated. One possibility is that respondents applied other stereotypes to Mehmet vignettes that affected their perceptions, such as the commonly held belief that Middle Eastern men are highly religious^31^. Future work should vary signals of religiosity in order to test this explanation. Another difference between the present research and prior work is the use of the lost wallet question as an outcome measure rather than a behavioral game or field experiment. It is possible that men of Turkish descent are thought to be more trustworthy in this specific scenario (returning lost goods) than in a more abstract or interdependent interaction like a trust game.

The second possibility is that respondents’ stated preferences do not match their real world expectations or behavior, i.e., that these results are affected by social desirability bias. Especially in an online survey where respondents have been exposed to other questions about migration, they may sense that the question relates to ethnic differences and respond in line with social norms not to discriminate by ethnicity. An important limitation of this research is that the GIP dataset does not yet include any measure of motivation to control prejudice. However, the present research is in several ways a least likely case for social desirability bias, at least when compared to other survey experimental approaches. First, online surveys are generally thought to be more resistant to such biases than survey modes employing an interviewer, as participants can answer questions anonymously and privately^53^. Second, I employ a between-subjects design where respondents see only a single vignette rather than seeing multiple vignettes that vary in potentially sensitive factors like ethnicity. This design decision was made to make the experimental treatment less obvious and thus reduce the potential for social desirability bias^54^. Finally, I signal ethnicity primarily through the visual cue of a photograph along with an ethnically typical name. Recent research finds that respondents generally discriminate more when provided with visual rather than textual (i.e., “he is German”) cues of ethnicity like those used here^55^. Future research should seek to determine the extent to which social desirability bias impacts survey responses about trustworthiness perceptions of outgroup members. It would also be informative to use other experimental methods that are less susceptible to social desirability, such as field experiments, to assess how appearance-related factors affect behavior in other settings.

Both explanations may be influenced by another factor that has become increasingly important in recent years: political polarization. Immigration has been widely discussed in German political debates since the arrival of hundreds of thousands of primarily Syrian refugees to the country in 2015. This extensive media coverage may have contributed to polarizing attitudes, driving moderates to more extreme views on the subject^56^, and thus more divergent trustworthiness perceptions. The hyperpartisan political environment may also affect respondents’ perceptions of what constitutes a socially desirable response, such that more exclusionary respondents are more likely to misreport their views^57^. This pattern is seemingly consistent with the results presented here, which show a marked preference for Turkish-origin vignette persons for inclusionary respondents and no ethnic differences for exclusionary respondents. If exclusionary respondents actually perceive the Mehmet vignettes to be less trustworthy, this would result in an underestimate of the ethnic trust gap. However, as stated above, my data do not allow me to assess the impact of social desirability bias on my results.

The present research has several other limitations. First, the photos used in the vignettes show only the face, unlike real-world conditions (such as a public place) where one would generally see the whole body. Thus, these results should be understood as the effects of facial attractiveness and not necessarily physical attractiveness. The photos vary only in terms of attractiveness and ethnicity, leaving out other potentially relevant factors like facial threat^58^ or face shape^59^, or perhaps most notably gender. Future research should assess whether attractiveness alters perceptions more strongly for female faces. Finally, as stereotypes may differ across national contexts, these results may not be generalizable to other countries. More research is needed in order to assess whether these trends hold elsewhere, and to determine what contextual factors might shape differential perceptions of trustworthiness.

Nevertheless, this project adds to the literature on the drivers of trustworthiness perceptions by exploring the interplay of ethnicity and attractiveness, two highly visible traits that are salient to first impressions. Such research on the role of intersectional stereotypes in shaping trustworthiness judgments remains rare, despite increasing recognition that intersectional social identities can significantly modify the content of ethnic stereotypes^31,60^. While it does not seem that physical attractiveness plays a substantial role in modifying ethnic biases in trustworthiness perceptions, other physical features or social identities might be more relevant. Additionally, findings about the role of immigration attitudes in shaping perceptions provide additional evidence that the intersection of vignette and respondent characteristics is important to understanding impression formation^11,61,62^. While examining such a wide variety of factors can be costly and complicated to implement, such research is needed in order to understand the complex interplay between trustor, trustee, and context that underlines trust-based interactions in diverse modern societies.

## Supplementary Information

Supplementary Information.

## Data Availability

Data from the German Internet Panel are available for scientific use upon application to the Leibniz Institute for the Social Sciences (GESIS), as described here: https://www.uni-mannheim.de/en/gip/for-data-users/ Replication code is available on OSF: https://osf.io/cg68r/
