# Supplementary material for: Facial attractiveness does not modify the perceived trustworthiness of ethnic minority men
Source: Sci Rep. 2024 Nov 7;14:27093. doi: 10.1038/s41598-024-78291-9 (PMC11544132; doi:10.1038/s41598-024-78291-9)
Supplement: Supplementary file 1 — Supplementary Information. [file 41598_2024_78291_MOESM1_ESM.pdf]

Supplementary Information
Facial attractiveness does not modify the perceived trustworthiness of ethnic minority men

Joshua Hellyer[1]

[1]Mannheim Centre for European Social Research, University of Mannheim

October 7, 2024

S1 Descriptive Statistics

	Selected	Unselected	Diff	pvalue
Male	0.52	0.53	0.01	0.74
Born 1970 or later	0.44	0.42	-0.02	0.25
Abitur	0.41	0.38	-0.03	0.04
In work or training	0.66	0.66	0.01	0.72
Resident of East Germany	0.21	0.20	-0.01	0.55
Living with partner	0.61	0.62	0.01	0.65
N	1794	1568		

Table S1: Means of demographic variables in the analytical sample ("Selected") and the rest of the sample ("Unselected") and t-test results.

S2 Supplemental Analyses: Main Results

	(1)<br>Model 1	(2)<br>Model 2	(3)<br>Model 3	(4)<br>Model 4	(5)<br>Model 5
High attractiveness	0.08*				
	(0.04)				
MENA phenotype x Mehmet		0.29***			
		(0.05)			
Ambig. phenotype x Mehmet			0.16**		
			(0.05)		
High att. x Jonas				0.05	
				(0.05)	
High att. x Mehmet					0.11*
					(0.05)
Constant	2.69***	2.56***	2.67***	2.59***	2.79***
	(0.03)	(0.03)	(0.04)	(0.03)	(0.04)
N	1794	895	899	900	894

Table S2: Full OLS regression results for main analyses without control variables, corresponding to results shown in Figure 2. Model 2 restricted to only profiles in unambiguous phenotype condition, Model 3 to only profiles with ambiguous phenotype. Model 4 restricted to only profiles in German ethnic condition, Model 5 to profiles in Turkish ethnic condition. Robust standard errors in parentheses, $^{+}p < .1$. $^{*}p < .05$. $^{**}p < .01$. $^{***}p < .001$.

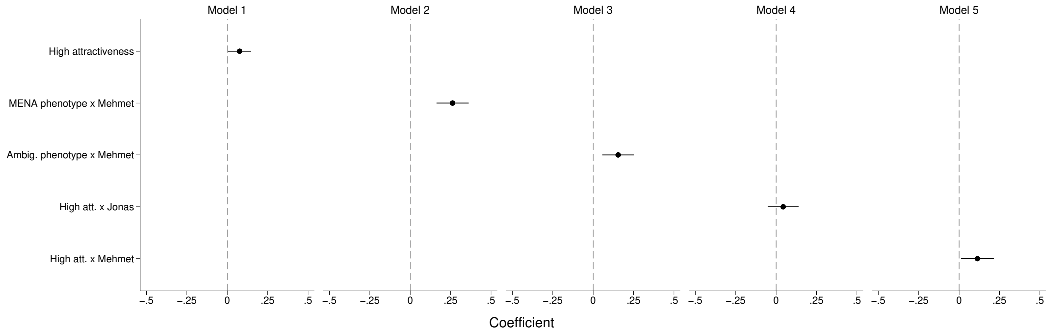

Figure S1: Coefficients and 95% confidence intervals from multivariate regression analysis (OLS) with control variables. Full sample, N = 1,794.

	(1) Model 1	(2) Model 2	(3) Model 3	(4) Model 4	(5) Model 5
High attractiveness	0.08^{*}				
	(0.04)				
MENA phenotype x Mehmet		0.26^{***}			
		(0.05)			
Ambig. phenotype x Mehmet			0.15^{**}		
			(0.05)		
High att. x Jonas				0.04	
				(0.05)	
High att. x Mehmet					0.11^{*}
					(0.05)
Abitur	0.13^{***}	0.16^{**}	0.09^{+}	0.05	0.20^{***}
	(0.04)	(0.05)	(0.05)	(0.05)	(0.05)
Male	-0.12^{**}	-0.08^{+}	-0.13^{**}	-0.05	-0.17^{***}
	(0.04)	(0.05)	(0.05)	(0.05)	(0.05)
Born 1970 or later	-0.17^{***}	-0.13^{*}	-0.22^{***}	-0.16^{*}	-0.19^{**}
	(0.04)	(0.06)	(0.06)	(0.06)	(0.06)
In work or training	-0.06	-0.10	0.01	-0.02	-0.10
	(0.05)	(0.06)	(0.07)	(0.07)	(0.07)
Lives in East Germany	-0.11^{*}	-0.06	-0.17^{**}	-0.10	-0.12^{+}
	(0.04)	(0.06)	(0.06)	(0.06)	(0.06)
Living with partner	0.15^{***}	0.15^{**}	0.12^{*}	0.17^{***}	0.10^{+}
	(0.04)	(0.05)	(0.05)	(0.05)	(0.06)
Constant	2.75^{***}	2.60^{***}	2.75^{***}	2.60^{***}	2.89^{***}
	(0.05)	(0.08)	(0.07)	(0.07)	(0.08)
N	1794	895	899	900	894

Table S3: Full OLS regression results for main analyses including control variables, corresponding to results shown in Figure S1. Model 2 restricted to only profiles in unambiguous phenotype condition, Model 3 to only profiles with ambiguous phenotype. Model 4 restricted to only profiles in German ethnic condition, Model 5 to profiles in Turkish ethnic condition. Robust standard errors in parentheses, $^{+}p < .1$. $^{*}p < .05$. $^{**}p < .01$. $^{***}p < .001$.

	(1) Model 1	(2) Model 2
High attractiveness	0.05	0.05
	(0.05)	(0.05)
Mehmet	0.20***	0.18***
	(0.05)	(0.05)
High att. x Mehmet	0.06	0.06
	(0.07)	(0.07)
Abitur		0.12***
		(0.04)
Male		-0.11**
		(0.04)
Born 1970 or later		-0.17***
		(0.04)
In work or training		-0.05
		(0.05)
Lives in East Germany		-0.11*
		(0.04)
Living with partner		0.13***
		(0.04)
Constant	2.59***	2.66***
	(0.03)	(0.06)
N	1794	1794

Table S4: Full OLS regression results testing the size of the beauty premium in trustworthiness across ethnic groups, with and without control variables. Robust standard errors in parentheses, $^{+}p < .1$. $^{*}p < .05$. $^{**}p < .01$. $^{***}p < .001$.

	(1) Model 1	(2) Model 2	(3) Model 3	(4) Model 4	(5) Model 5
High attractiveness	0.20* (0.09)				
MENA phenotype x Mehmet		0.65*** (0.13)			
Ambig. phenotype x Mehmet			0.42*** (0.13)		
High att. x Jonas				0.11 (0.13)	
High att. x Mehmet					0.29* (0.13)
Abitur	0.31*** (0.09)	0.37** (0.13)	0.19 (0.13)	0.12 (0.13)	0.44*** (0.12)
Male	-0.28** (0.09)	-0.21 (0.13)	-0.32* (0.13)	-0.14 (0.13)	-0.41** (0.13)
Born 1970 or later	-0.45*** (0.11)	-0.34* (0.15)	-0.60*** (0.17)	-0.40* (0.17)	-0.48** (0.16)
In work or training	-0.16 (0.12)	-0.26 (0.16)	0.03 (0.17)	-0.08 (0.17)	-0.23 (0.16)
Lives in East Germany	-0.29** (0.11)	-0.17 (0.16)	-0.41* (0.16)	-0.28+ (0.16)	-0.29+ (0.16)
Living with partner	0.35*** (0.09)	0.35* (0.14)	0.33* (0.13)	0.45*** (0.13)	0.23+ (0.14)
cut1	-5.44*** (0.36)	-5.32*** (0.54)	-5.29*** (0.49)	-5.77*** (0.61)	-5.25*** (0.46)
cut2	-3.64*** (0.19)	-3.34*** (0.28)	-3.66*** (0.26)	-3.34*** (0.25)	-4.02*** (0.29)
cut3	-0.50*** (0.13)	-0.17 (0.20)	-0.50** (0.18)	-0.19 (0.18)	-0.84*** (0.20)
cut4	1.66*** (0.14)	1.98*** (0.20)	1.72*** (0.19)	2.15*** (0.20)	1.25*** (0.20)
N	1794	895	899	900	894

Table S5: Full ordered logistic regression results for main analyses including control variables. Model 2 restricted to only profiles in unambiguous phenotype condition, Model 3 to only profiles with ambiguous phenotype. Model 4 restricted to only profiles in German ethnic condition, Model 5 to profiles in Turkish ethnic condition. Robust standard errors in parentheses, $^{+}p < .1$. $^{*}p < .05$. $^{**}p < .01$. $^{***}p < .001$.

	(1) Model 1	(2) Model 2	(3) Model 3	(4) Model 4
Pre-rated attractiveness	-0.01		0.01	0.01
	(0.03)		(0.04)	(0.04)
Mehmet			0.09	0.03
			(0.36)	(0.35)
Pre-rated att. x Jonas		-0.00		
		(0.03)		
Pre-rated att. x Mehmet		0.05	0.04	0.04
		(0.03)	(0.06)	(0.06)
Abitur				0.16**
				(0.05)
Male				-0.08
				(0.05)
Born 1970 or later				-0.13*
				(0.06)
In work or training				-0.10
				(0.07)
Living with partner				0.15**
				(0.05)
Lives in East Germany				-0.05
				(0.06)
Constant	2.77***	2.56***	2.52***	2.57***
	(0.18)	(0.18)	(0.23)	(0.23)
N	895	895	895	895

Table S6: Full OLS regression results testing the size of the beauty premium in trustworthiness across ethnic groups using continuous measures of facial attractiveness from pre-test ratings. Analyses limited to unambiguous faces (i.e., those used with only one name) to more strictly test the effects of appearance rather than name. Robust standard errors in parentheses, $^{+}p < .1$. $^{*}p < .05$. $^{**}p < .01$. $^{***}p < .001$.

	(1) Model 1	(2) Model 2	(3) Model 3	(4) Model 4	(5) Model 5
High attractiveness	0.08^{+} (0.04)				
MENA phenotype x Mehmet		0.25^{***} (0.06)			
Ambig. phenotype x Mehmet			0.13^{*} (0.06)		
High att. x Jonas				0.01 (0.06)	
High att. x Mehmet					0.13^{*} (0.06)
Abitur	0.14^{***} (0.04)	0.17^{**} (0.06)	0.10^{+} (0.06)	0.06 (0.06)	0.22^{***} (0.06)
Male	-0.16^{***} (0.04)	-0.13^{*} (0.06)	-0.17^{**} (0.06)	-0.13^{*} (0.06)	-0.19^{**} (0.06)
Born 1970 or later	-0.14^{**} (0.05)	-0.06 (0.07)	-0.23^{**} (0.07)	-0.16^{*} (0.07)	-0.10 (0.07)
In work or training	-0.06 (0.05)	-0.12 (0.07)	0.01 (0.07)	-0.02 (0.07)	-0.11 (0.07)
Lives in East Germany	-0.13^{*} (0.06)	-0.03 (0.08)	-0.23^{**} (0.08)	-0.12 (0.07)	-0.14^{+} (0.08)
Living with partner	0.11^{*} (0.04)	0.13^{*} (0.06)	0.09 (0.06)	0.11^{+} (0.06)	0.10 (0.07)
Lives in urban area	-0.09^{+} (0.05)	-0.16^{*} (0.08)	-0.03 (0.07)	-0.09 (0.07)	-0.10 (0.08)
Pct. migrants in community	-0.00 (0.00)	0.00 (0.01)	-0.01 (0.01)	-0.01^{+} (0.01)	0.01 (0.01)
Constant	2.89^{***} (0.07)	2.73^{***} (0.11)	2.91^{***} (0.10)	2.88^{***} (0.11)	2.91^{***} (0.11)
N	1370	677	693	671	699

Table S7: Full OLS regression results including controls related to respondents' location of residence, for respondents with valid postal code. Model 2 restricted to only profiles in unambiguous phenotype condition, Model 3 to only profiles with ambiguous phenotype. Model 4 restricted to only profiles in German ethnic condition, Model 5 to profiles in Turkish ethnic condition. "Urban area" defined as a community of 10,000 residents or more. Percentage of migrants and community population at the postal code level derived from 2011 Zensus data. Robust standard errors in parentheses, $^{+}p < .1$. $^{*}p < .05$. $^{**}p < .01$. $^{***}p < .001$.

S3 Supplemental Analyses: Heterogeneous Effects

S3.1 Immigration Attitudes

	(1)<br>Model 1a	(2)<br>Model 1b	(3)<br>Model 2a	(4)<br>Model 2b
Jonas x inclusionary	0.00<br>(.)	0.16**<br>(0.06)		
Jonas x exclusionary	-0.16**<br>(0.06)	0.00<br>(.)		
Mehmet x inclusionary	0.26***<br>(0.04)	0.43***<br>(0.06)		
Mehmet x exclusionary	-0.21**<br>(0.08)	-0.04<br>(0.09)		
Low att. x Mehmet x inclusionary (ref.)			0.00<br>(.)	
High att. x Mehmet x inclusionary			0.12*<br>(0.06)	
Low att. x Mehmet x exclusionary (ref.)				0.00<br>(.)
High att. x Mehmet x exclusionary				0.03<br>(0.14)
Constant	2.67***<br>(0.03)	2.51***<br>(0.05)	2.87***<br>(0.04)	2.45***<br>(0.10)
N	1638	1638	652	160

Table S8: Full OLS regression results for analyses divided by immigration attitudes, corresponding to results shown in Figure 3. Robust standard errors in parentheses, $^{+}p < .1$. $^{*}p < .05$. $^{**}p < .01$. $^{***}p < .001$.

S3.2 Mosque Support

	(1) Model 1a	(2) Model 1b	(3) Model 2a	(4) Model 2b
Jonas x pro-mosque	0.00	0.03		
	(.)	(0.05)		
Jonas x anti-mosque	-0.03	0.00		
	(0.05)	(.)		
Mehmet x pro-mosque	0.36***	0.40***		
	(0.05)	(0.05)		
Mehmet x anti-mosque	0.03	0.07		
	(0.06)	(0.06)		
Low att. x Mehmet x pro-mosque (ref.)			0.00	
			(.)	
High att. x Mehmet x pro-mosque			0.11	
			(0.07)	
Low att. x Mehmet x anti-mosque (ref.)				0.00
				(.)
High att. x Mehmet x anti-mosque				0.04
				(0.09)
Constant	2.66***	2.62***	2.96***	2.67***
	(0.04)	(0.04)	(0.05)	(0.05)
N	1540	1540	369	395

Table S9: Full OLS regression results for analyses divided by support or opposition to mosque construction. Robust standard errors in parentheses, $^{+}p < .1$. $^{*}p < .05$. $^{**}p < .01$. $^{***}p < .001$.

	(1) Model 1	(2) Model 2	(3) Model 3	(4) Model 4
Mehmet	0.26***	0.25***	0.36***	0.35***
	(0.04)	(0.04)	(0.05)	(0.05)
Exclusionary attitudes	-0.16**	-0.13*		
	(0.06)	(0.06)		
Exclusionary x Mehmet	-0.30**	-0.29**		
	(0.10)	(0.10)		
Opposes mosque			-0.03	-0.03
			(0.05)	(0.05)
Opposes mosque x Mehmet			-0.29***	-0.29***
			(0.08)	(0.08)
Male		-0.10**		-0.15***
		(0.04)		(0.04)
Born 1970 or later		-0.18***		-0.16**
		(0.05)		(0.05)
Abitur		0.05		0.09*
		(0.04)		(0.04)
In work or training		-0.00		-0.04
		(0.05)		(0.05)
Lives in East Germany		-0.08+		-0.10*
		(0.05)		(0.05)
Living with partner		0.11**		0.13**
		(0.04)		(0.04)
Constant	2.67***	2.73***	2.66***	2.74***
	(0.03)	(0.06)	(0.04)	(0.06)
N	1638	1638	1540	1540

Table S10: Full OLS regression results testing the extent of ethnic bias in trustworthiness perceptions across groups defined by immigration attitudes and attitudes toward Islam, with and without control variables. Robust standard errors in parentheses, $^{+}p < .1$. $^{*}p < .05$. $^{**}p < .01$. $^{***}p < .001$.

S3.3 Respondent Gender

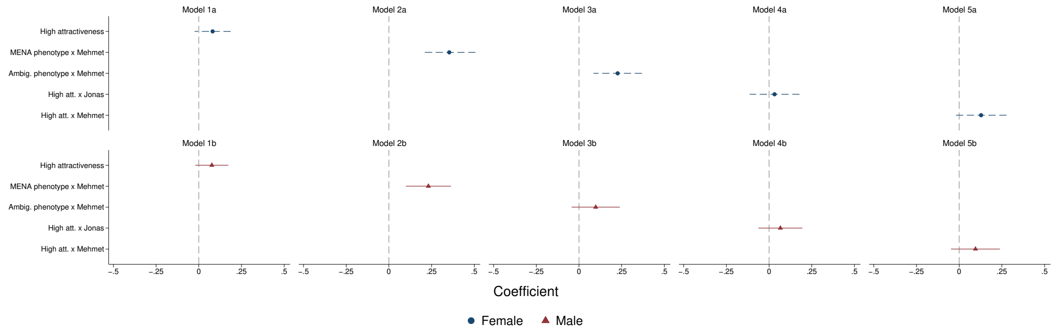

Figure S2: Coefficients and 95% confidence intervals from multivariate regression analysis (OLS) without control variables. Sample divided into male (n = 937) and female respondents (n = 857).

	(1) Model 1a	(2) Model 2a	(3) Model 3a	(4) Model 4a	(5) Model 5a
High attractiveness	0.08				
	(0.05)				
MENA phenotype x Mehmet		0.35^{***}			
		(0.08)			
Ambig. phenotype x Mehmet			0.23^{**}		
			(0.07)		
High att. x Jonas				0.03	
				(0.07)	
High att. x Mehmet					0.13^{+}
					(0.08)
Constant	2.74^{***}	2.57^{***}	2.69^{***}	2.62^{***}	2.86^{***}
	(0.04)	(0.06)	(0.05)	(0.05)	(0.05)
N	857	408	449	425	432

Table S11: Full OLS regression results for female respondents only, corresponding to results shown in Figure S2. Robust standard errors in parentheses, $^{+}p < .1$. $^{*}p < .05$. $^{**}p < .01$. $^{***}p < .001$.

	(1) Model 1b	(2) Model 2b	(3) Model 3b	(4) Model 4b	(5) Model 5b
High attractiveness	0.08				
	(0.05)				
MENA phenotype x Mehmet		0.23^{***}			
		(0.07)			
Ambig. phenotype x Mehmet			0.10		
			(0.07)		
High att. x Jonas				0.07	
				(0.07)	
High att. x Mehmet					0.10
					(0.07)
Constant	2.64^{***}	2.55^{***}	2.65^{***}	2.56^{***}	2.72^{***}
	(0.04)	(0.04)	(0.05)	(0.05)	(0.05)
N	937	487	450	475	462

Table S12: Full OLS regression results for male respondents only, corresponding to results shown in Figure S2. Robust standard errors in parentheses, $^{+}p < .1$. $^{*}p < .05$. $^{**}p < .01$. $^{***}p < .001$.

S3.4 Socioeconomic Status

	(1) Model 1a	(2) Model 1b	(3) Model 2a	(4) Model 2b
Jonas x no Abitur	0.00	-0.01	0.00	-0.06
	(.)	(0.05)	(.)	(0.05)
Jonas x Abitur	0.01	0.00	0.06	0.00
	(0.05)	(.)	(0.05)	(.)
Mehmet x no Abitur	0.17^{***}	0.16^{**}	0.15^{**}	0.10^{+}
	(0.05)	(0.05)	(0.05)	(0.05)
Mehmet x Abitur	0.31^{***}	0.29^{***}	0.34^{***}	0.29^{***}
	(0.05)	(0.05)	(0.05)	(0.05)
Male			-0.11^{**}	-0.11^{**}
			(0.04)	(0.04)
Born 1970 or later			-0.17^{***}	-0.17^{***}
			(0.04)	(0.04)
In work or training			-0.05	-0.05
			(0.05)	(0.05)
Lives in East Germany			-0.11^{**}	-0.11^{**}
			(0.04)	(0.04)
Living with partner			0.14^{***}	0.14^{***}
			(0.04)	(0.04)
Constant	2.61^{***}	2.62^{***}	2.71^{***}	2.77^{***}
	(0.03)	(0.04)	(0.05)	(0.06)
N	1794	1794	1794	1794

Table S13: Full OLS regression results testing the extent of ethnic bias in trustworthiness perceptions across groups defined by educational attainment (having received or not received the *Abitur*, or German matriculation examination), with and without control variables. Robust standard errors in parentheses, $^{+}p < .1$. $^{*}p < .05$. $^{**}p < .01$. $^{***}p < .001$.

	(1) Model 1a	(2) Model 1b	(3) Model 2a	(4) Model 2b
Pre-rated social class	0.03	-0.10	0.04	-0.09
	(0.07)	(0.09)	(0.07)	(0.09)
Mehmet	0.18^{***}	0.27^{***}	0.16^{**}	0.26^{***}
	(0.05)	(0.06)	(0.05)	(0.06)
Male			-0.18^{***}	-0.00
			(0.05)	(0.05)
Born 1970 or later			-0.19^{***}	-0.14^{*}
			(0.06)	(0.07)
In work or training			-0.11^{+}	0.07
			(0.06)	(0.07)
Lives in East Germany			-0.15^{**}	-0.06
			(0.06)	(0.07)
Living with partner			0.13^{*}	0.15^{**}
			(0.05)	(0.06)
Constant	2.41^{***}	3.23^{***}	2.53^{***}	3.15^{***}
	(0.43)	(0.54)	(0.42)	(0.54)
N	1051	743	1051	743

Table S14: Full OLS regression results for analyses divided by respondent educational attainment (having received or not received the *Abitur*, or German matriculation examination). Models 1a and 2a include only respondents without *Abitur*, models 1b and 2b include only respondents with *Abitur*. Robust standard errors in parentheses, $^{+}p < .1$. $^{*}p < .05$. $^{**}p < .01$. $^{***}p < .001$.
